# Nice guideline on thyroid disease: where does it take us with liothyronine?

**DOI:** 10.1186/s13044-020-00081-y

**Published:** 2020-05-23

**Authors:** Graham P. Leese

**Affiliations:** Ward 5 Ninewells Hospital, Dundee, DD1 9SY UK

**Keywords:** Liothyronine, L-thyroxine, Thyroid, Guidelines

## Abstract

The new NICE guidelines on thyroid disease and its management do not recommend the routine use of liothyronine, but do not completely rule it out either. Guidelines from the British and European Thyroid Associations are open to a “trial of liothyronine” on an individual basis.

Some patients do not feel well on L-thyroxine despite a serum TSH in the reference range. Key issues to consider in such patients include establishing whether the patient had established hypothyroidism initially, and whether the L-thyroxine has been titrated carefully enough, possibly using small increments, to achieve a careful balance between symptoms and serum TSH concentrations. Patients should also be considered for other causes of the symptoms which may be wide-ranging.

Meta-analyses of several, but small, randomised control trials show no advantage, or disadvantage of liothyronine over L-thyroxine. However, detailed sub-analysis identifies some tantalising results eg on preferential weight loss, patient preference, and possibly genetic markers. Although linked with plausible theoretical explanations, these results may be over-interpreted. The key questions are whether a short-term trial treatment is worthwhile and safe, and whether in the future sub-groups of patients can be identified who may benefit from liothyronine. These questions remain divisive but require additional focussed research.

It could be argued that inflated costs of liothyronine in some countries have either distracted from or helped focus on the science. Costs need to be addressed. However better biomarkers of tissue level thyroid action, and a better understanding of the impact of genetic polymorphisms will help to make progress when choosing if there is a place for liothyronine in the future.

(words: 262)

## Background

The debate on whether liothyronine has a role in managing hypothyroidism is akin to entering a cauldron where there is reasonably clear, but possibly weak, clinical evidence but with intriguing and tantalising questions in the detail, alongside a strong patient lobby, Pharmaceutical financial opportunism and Politicians who are conflicted between a popularist approach and a need for fiscal responsibility in health-care.

### Sorting L-thyroxine prescribing first of all

Titrating the correct dose of L-thyroxine for patients with primary hypothyroidism is usually straight-forward but can be challenging in some patients, as around 5–10% of patients on L-thyroxine have persistent symptoms after starting treatment [1, 2]. The goal is to achieve a balance between symptomatic improvement and maintaining a serum thyroid-stimulating hormone (TSH) concentration in the reference range so as to avoid adverse events [3]. Several patients do seem to prefer a dose of L-thyroxine that results in a serum TSH in the lower part of the reference range, and some even with a serum TSH below the reference range. The availability of 12.5 microgram dose strengths of L-thyroxine makes this titration easier. However, patients are increasingly being started on L-thyroxine at more marginal baseline serum TSH concentrations [4] often with transient or marginally abnormal serum TSH where hypothyroidism is unlikely to be the cause of their symptoms. We now realise that the reference range should probably be expanding, especially in older patients [5]. Some patients do not gain persistent benefit with increasing doses of L-thyroxine despite achieving a low or even suppressed TSH. Thus careful consideration of many other potential causes of their symptoms should be considered, including important conditions such as hypoadrenalism and anaemia. However, co-prescribed drugs, sleep apnoea, chronic fatigue, stressful lifestyles are all more common causes of symptoms that may be mistaken for non-specific hypothyroid symptoms.

The new National Institute for Clinical Excellence (NICE) guidelines [6] recommend using L-thyroxine, with no routine use of liothyronine (section 1.3.4). For healthy people under the age of 65 a dose of 1.6microgram/kg body weight/day is advised from the start, whilst those over 65 years or with a history of cardiovascular disease should be started on 25 or 50 microgram of L-thyroxine. The dose should be adjusted to achieve a serum TSH within the reference range, acknowledging that the serum TSH can take some time to change. Most clinicians will adjust the dose of thyroxine being guided by both symptom control and serum TSH. This acknowledges that a number of different L-thyroxine doses may be compatible with a serum TSH in the reference range.

## Main text

### Is there a role for Liothyronine?

The NICE guidelines [6] do comment that some patients who do not feel well on L-thyroxine alone “are sometimes offered liothyronine”. The guideline does not give a clear recommendation to use Liothyronine but imply that there may be a role for liothyronine for patients with adverse symptoms despite seemingly adequate L-thyroxine replacement, and that more research is required. The British Thyroid Association [7] and European Thyroid Association [8] provide clearer guidance. For patients not achieving adequate symptom control on L-thyroxine where other potential causes of the symptoms have been excluded, they suggest that Liothyronine in combination with L-thyroxine may be considered by a specialist Endocrinologist on a trial basis whilst maintaining the serum TSH in the reference range. The trial of liothyronine should be stopped after 3–4 months if there is no patient benefit. However, across the UK the approach to liothyronine prescribing has been very variable. In some areas it has proved virtually impossible to access it, in others there is a fairly restricted approach, whilst in a decreasing number of areas it has been more freely available.

### Is Liothyronine beneficial or harmful compared to L-thyroxine?

Meta-analyses have compared liothyronine use (alone or in combination with L-thyroxine) against L-thyroxine alone, but demonstrated no consistent benefit for liothyronine use over L-thyroxine alone [6, 9, 10]. Unfortunately, only three of the original studies included more than 100 patients, and many are under powered. It has been concluded that liothyronine demonstrates no overall benefit and in addition there may be risks. Current forms of oral liothyronine cause transient non-physiological elevated serum tri-iodothyronine concentrations post-dosing [11], which are not thought to result in low serum TSH concentrations but may still be harmful. More flexible dosing regimes of liothyronine aided by the introduction of 5 microgram tablets, could make liothyronine dosing easier and potentially safer if they reduced the risk of raised serum tri-iodothyronine, although they are unlikely to eliminate this risk. Other options would include the use of a slow-release preparation of liothyronine which is being explored.

These same meta-analyses [6, 9, 10] could be interpreted that liothyronine is not inferior to L-thyroxine, and the lack of difference in adverse events [9] as reassuring. A 17 year population-based observational follow up study with 400 people on liothyronine also showed no increase risk of fractures, atrial fibrillation or cardiovascular disease [12] with patients achieving a mean serum TSH of 1.1 mU/L during follow up, although there was an increased risk of prescription for incident psychotropic medication. Intriguingly some studies showed marginally enhanced weight loss (~ 1.5 kg) on a liothyronine/L-thyroxine combination compared to L-thyroxine alone, whilst patients were maintaining a serum TSH in the reference range [13, 14]. Also in five cross-over studies more patients preferred combination therapy rather than L-thyroxine alone (48% vs 27% - [8]). It is thus possible that there is a subset of patients who may benefit from liothyronine. Is there a potential rationale for this or are these findings due to random chance?

L-thyroxine replacement results in higher serum free thyroxine (T4) concentrations which suppresses deiodinase activity and lowers serum T3 concentrations. Rat studies required a combination of thyroxine and liothyronine replacement to achieve physiological tissue serum concentrations [15]. However, similar studies in humans did not seem to result in improved patient reported outcomes [8, 16] and in another study serum T4 and serum triodothyronine (T3) concentrations were no different between self-reported “responders” and non-responders” to liothyronine therapy [17]. This may be because intracellular concentrations of T3 are dependent on a huge number of factors, including membrane transport proteins, de-iodination, nuclear protein binding e.g. with retinoid X receptor (RXR) and thyroid hormone receptors, which are all downstream from serum concentrations of thyroid hormones. Hence measuring serum free thyroxine and liothyronine may not be the way to understand this enigma and we may need novel biomarkers . Intriguingly other biomarkers have been used in some studies, and despite similar serum TSH concentrations, use of liothyronine resulted in lower serum cholesterol, higher sex hormone binding globulin (SHBG) and increased markers of bone turnover [18–20]. We cannot however ignore serum TSH, which we do know is a useful marker of long-term patient safety of fractures and cardiovascular disease [3]. It may be a blunt marker however with risks increasing significantly only when the serum TSH was below 0.1 mU/l, despite the lower limit of the reference range being 0.4 mU/L.

The main enzyme converting T4 to T3 in tissues is deiodinase 2 (DIO2). Thus the initial studies showing that a polymorphism on the DIO2 gene was associated with a better response to liothyronine than the wild type [2] was intuitive and exciting. However further studies have not corroborated this finding in follow up [21]. Other polymorphisms on the monocarboxylate transporter 10 (MCT10) thyroid transporter protein, especially in combination with DIO2 polymorphisms have also been potentially implicated [22]. Genetic epidemiology is however fraught with false dawns, especially when studying candidate genes and much larger study cohorts with replication studies, and GWAS studies are required in this area before any conclusions can be drawn.

### Costs of Liothyronine

The astronomical increase in costs of liothyronine over the last 10 years in the UK has resulted in a focus on the benefits and risks of liothyronine that probably would otherwise have received less attention given the age of the drug liothyronine. With the price of liothyronine 20microgram peaking at around £250 per month in the UK, and following parliamentary activity from the House of Lords, the number of suppliers has increased from one to three and the price is starting to fall, currently being available at £174 per month (at the time of writing). However this compares with a cost of around £4 ten years ago, a cost of about £6 per month in Germany and an equivalent dose cost of around £1 per month for L-thyroxine. This has also resulted in post-code prescribing, with patients in the most deprived parts of England being half as likely to receive a prescription for liothyronine as people in the most affluent areas [23].

### Pragmatic approach for now

Thus in summary there is no robust data that liothyronine is of benefit in patients requiring thyroid replacement, which is why guidelines recommend that the vast majority of patients should be treated with L-thyroxine. However there are patient reports of benefit and some intriguing data which may support this. There appears to be no apparent major risks of using liothyronine as long as the serum TSH is maintained within the reference range. Thus careful judicious use of liothyronine use may be warranted in a few patients who continue to report adverse symptoms that cannot be attributed to another cause [7, 8]. Using a combination of L-thyroxine and liothyronine avoids the need for high doses of liothyronine, and may thus avoid the concerns of hyperthyroxinaemia, although it seems this cannot be eliminated with current formulations [11]. Most studies use a ratio of 1:5 or 1:4 for Liothyronine to L-thyroxine, but some have used a 1:3 or a 1:10 ratio. There is no clear consensus about equivalent doses of L-thyroxine and Liothyronine but most clinicians reduce the former by 50microgram and introduce 10–20 microgram of liothyronine [16]. A long acting preparation of liothyronine is also being explored with the consideration of it being safer.

### What do we need to improve the situation for patients?

Firstly we need to address inappropriate initiation, or continuation of L-thyroxine. When patients are started on L-thyroxine inappropriately it is not surprising that they may not feel better on treatment. There is a great desire to address a patient’s feelings of lack of well being, and coupled with the often non-specific symptoms that can be associated with hypothyroidism and in the presence of a borderline serum TSH result it is understandable to consider treating with L-thyroxine. Thus, for individuals in such circumstances it may reasonable to start a “trial” of L-thyroxine for around three months. Sometimes there can be an immediate benefit but if the clinical benefit is not maintained then treatment should not be continued in patients with a baseline borderline serum TSH, as otherwise it raises unrealistic expectations about the role of L-thyroxine for them in the long-term.

Secondly we need better biomarkers to measure the impact of thyroid replacement therapy. Serum TSH is probably the best one we have currently, but serum free-T4 and serum free-T3 concentrations in general do not reflect the complex intracellular homeostasis of thyroid physiology.

Most of the randomised controlled clinical trials of liothyronine are too small to address some of the clinical endpoints that are being measured. Some trials may include patients where the initial diagnosis of hypothyroidism was questionable but have been established on L-thyroxine as explained above. Future trials of liothyronine should only include patients where the diagnosis of hypothyroidism is robust, such as having a TSH of greater than 10 mU/L or a serum free T4 below the reference range prior to diagnosis. However, the current trials identify some intriguing results on the use of liothyronine e.g. weight changes and genetic studies, which suggest that some subgroups may benefit more than others, but the trials are too small to draw useful conclusions. Also it may be best to focus research on patients who feel unwell on L-thyroxine rather than including *all* patients with hypothyroidism [6]. In addition as there is a suggestion of variable tissue level effects eg brain versus liver, further exploration of the differential impact of liothyronine on symptoms eg brain fog versus fatigue, may be warranted.

Fourthly, the issue of pricing for liothyronine in the UK needs to be addressed. Why has the price been allowed to increase so much in the last 10 years? Why is the price so much more than other European countries e.g. more than 30-fold greater? Why has it not decreased more since the introduction of multiple suppliers and the breaking of the previous monopoly? There is a need for procurement teams to negotiate better deals.

## Conclusion

There is a sense that the issues surrounding the use of liothyronine are like a storm in a teacup whipped up by the winds of pricing. However there are real issues about benefit and risk which remain scientifically unresolved unfortunately. A pragmatic approach has been outlined, but in the future we need to move towards identifying if there are any subgroups of hypothyroid patients who may benefit from using liothyronine in addition to L-thyroxine, probably by identifying novel biomarkers or genetic polymorphisms.

## Data Availability

There is no original data and no materials available.
